# Programmed cell death in spinal cord injury pathogenesis and therapy

**DOI:** 10.1111/cpr.12992

**Published:** 2021-01-27

**Authors:** Zhongju Shi, Shiyang Yuan, Linlin Shi, Jiahe Li, Guangzhi Ning, Xiaohong Kong, Shiqing Feng

**Affiliations:** ^1^ Department of Orthopaedics Tianjin Medical University General Hospital Tianjin China; ^2^ School of Medicine Nankai University Tianjin China; ^3^ Tianjin Key Laboratory of Spine and Spinal Cord International Science and Technology Cooperation Base of Spinal Cord Injury Tianjin China

**Keywords:** pathological mechanisms, programmed cell death, spinal cord injury, therapy

## Abstract

Spinal cord injury (SCI) always leads to functional deterioration due to a series of processes including cell death. In recent years, programmed cell death (PCD) is considered to be a critical process after SCI, and various forms of PCD were discovered in recent years, including apoptosis, necroptosis, autophagy, ferroptosis, pyroptosis and paraptosis. Unlike necrosis, PCD is known as an active cell death mediated by a cascade of gene expression events, and it is crucial for elimination unnecessary and damaged cells, as well as a defence mechanism. Therefore, it would be meaningful to characterize the roles of PCD to not only enhance our understanding of the pathophysiological processes, but also improve functional recovery after SCI. This review will summarize and explore the most recent advances on how apoptosis, necroptosis, autophagy, ferroptosis, pyroptosis and paraptosis are involved in SCI. This review can help us to understand the various functions of PCD in the pathological processes of SCI, and contribute to our novel understanding of SCI of unknown aetiology in the near future.

## Graphical abstract

Different types of programmed cell death (PCD) play different roles in spinal cord injury. PCD is an active cell death mediated by a cascade of gene expression events, and it is crucial for elimination unnecessary and damaged cells, as well as a defense mechanism. PCD consists of a series of activities, such as apoptosis, necroptosis, autophagy, ferroptosis, pyroptosis and paraptosis. This figure indicates how how apoptosis, necroptosis, autophagy, ferroptosis, pyroptosis and paraptosis are involved in spinal cord injury.

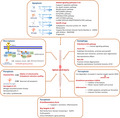

## 1 INTRODUCTION

Spinal cord injury (SCI) usually results in a large range of sensorimotor and autonomic nerve injury and remains a serious public health problem worldwide. SCI affects approximately 273 000 people in the United States, and there are some 12 000 new cases each year.^1^, ^2^, ^3^ Therefore, SCI brings severe economy burdens and psychological pressure to patients. However, there are currently no effective therapies for SCI clinically, and an effective treatment is awaited.^4^, ^5^, ^6^ This is due mainly to the molecular mechanisms of SCI remain elusive. The pathological process of SCI is known as a complex process, which can be classified into two phases: Primary injury is the direct mechanical damage of spinal cord tissue and includes demyelination and necrosis of neurons and axons; and the secondary injury is composed of a variety of pathophysiologic mechanisms, including local haemorrhage, ischaemia, oedema, ionic imbalance, free radical stress and inflammatory responses.^7^, ^8^ This complex pathological process of SCI may explain the difficulty in finding a suitable and effective therapy. Therefore, understanding the molecular mechanisms of SCI is critical for the development of therapeutic strategies.

Cell death is known as the final stage of cells and it can be resulted from cytotoxicity from exogenous or endogenous substances.^9^ In 1842, cell death was first posed by Carl Vogt, and lots of molecules are considered to be involved in this irreversible process to support the maintenance of cellular homeostasis.^10^ Cell death was initially divided into two types, necrosis and apoptosis.^11^ Necrosis is considered as a passive and accidental cell death, which can be resulted from environmental perturbations and the large amounts of release of inflammatory cellular contents.^11^, ^12^ Apoptosis is considered as an active, programmed process of autonomous cellular dismantling without the release of cytoplasmic content to the extracellular space.^11^, ^12^, ^13^, ^14^ In recent years, various forms of programmed cell death (PCD) were discovered, including autophagy, necroptosis, pyroptosis and ferroptosis.^9^, ^15^, ^16^, ^17^, ^18^ Unlike necrosis, PCD is known as an active cell death mediated by a cascade of gene expression events, commonly found in organisms.^15^, ^16^ PCD is important for elimination unnecessary and damaged cells, and it can also represent a defence mechanism.^19^, ^20^, ^21^ As research has progressed, more and more researches showed that PCD was involved in central nervous system (CNS) development, neurodegenerative disorders, psychiatric disorders and CNS injury.^22^

More recently, there still is a lack of the study on PCD in SCI, and increasing evidence shows that PCD play significant roles following SCI.^23^, ^24^ In this review, we summarized and explored the recent advances in the roles of PCD in SCI in order to know the molecular mechanisms underlying SCI.

## 2 THE ROLE OF APOPTOSIS IN SPINAL CORD INJURY

As a type of PCD, apoptosis is the most common form of PCD, it was extensively studied and could be defined as programmed self‐killing simply.^25^ Apoptosis has been considered as an immunologically silent form of PCD since cell corpses are cleared by phagocytes, and it can arise from the extrinsic (death receptor initiated) and the intrinsic (mitochondrial) pathways.^26^, ^27^, ^28^ Excitotoxins, free radicals and inflammatory mediators are known as the factors that initiate cell death and stimulate necrosis or apoptosis.^29^ Apoptosis is known as a naturally occurring physiological process and may play a key role in secondary SCI.^29^ The secondary injury following SCI is considered to be due to the continuation of cellular destruction through apoptosis, and the long‐term neurological deficits following SCI might result from a large range of apoptosis of neurons and oligodendrocytes in the injured spinal cord, so a better understanding of apoptosis following SCI will lead to novel strategies for therapeutic interventions.

Apoptosis can be found in the injured spinal cord of animal models and humans. Injury‐induced apoptosis could be observed in neurons, astrocytes, oligodendroglia and microglia in rat spinal cord after injury,^30^ and the apoptotic oligodendrocytes mainly located in the longitudinal tracts of the white matter.^31^ Apoptosis in neurons was detected at 4 hours and peaked at 8 hours after injury; apoptosis in glial cells was detected at 4 hours after injury, and peaked at 24 hours after injury; apoptosis in oligodendrocytes was detected in the white matter at 24 hours and reached to the highest at 8 days after injury.^32^, ^33^ Apoptosis in microglia was relatively rare at 24 hours and 5 days, but gradually increased in numbers and peaked at 8 days post‐injury.^33^ Furthermore, most of apoptotic cells could be observed in the rim of surviving tissue around the centre of the injured spinal cord, and this may help explain why the lesion area expand, progressively.^29^ Apoptosis can also be detected in oligodendrocytes in white matter tracts showing Wallerian degeneration, which is a significant characteristic in chronic phase of SCI.^29^, ^34^

In recent years, more and more studies occur to investigate the role of apoptosis after SCI. Apoptosis is a caspase‐dependent cell death modality, and this programme was primarily regulated by the caspase family of cysteine proteases.^35^ Upstream and downstream components of the caspase‐3 apoptotic pathway have proved to be activated after SCI in rats and play a vital role in the process of apoptosis.^36^ Previous studies showed that activation of the Fas death receptor pathway played a key role in neuronal, oligodendrocyte and microglia apoptosis in animal models following SCI.^37^, ^38^, ^39^ The interaction of Fas and Fas receptor (FasR) initiates apoptosis via the activation of the cysteine proteases resulting in proteolysis and DNA cleavage by effector caspases culminating in cell death.^40^, ^41^ Some key signalling pathways, including SIRT1/AMPK signalling pathway, Wnt/β‐catenin signalling pathway and E2F1/CDK1 pathway, have already proved to be involved in regulating apoptotic activity in SCI.^42^, ^43^, ^44^ Furthermore, recent studies revealed that microRNAs (miRNAs) and long non‐coding RNAs (lncRNAs) may have an important effect on the pathogenesis of SCI including cell apoptosis. A previous study revealed that miR‐137 could inhibit apoptosis after SCI via targeting MK2.^45^ Gu et al^46^ found that knockdown of long coding RNA XIST played a critical role in inhibiting neuronal apoptosis after SCI by competitively binding miR‐494 and regulation on PTEN/AKT/mTOR pathway. Another study showed that PI3K/Akt/mTOR signalling pathway took part in the apoptosis of neurons after SCI and this pathway could induce apoptosis by activation of the mitochondrial pathway.^47^

Having an in‐depth knowledge of the molecular and cellular mechanisms of apoptosis can help us screen out the specific drug targets. Liu et al used proteomics analysis to find a key protein, endoplasmic reticulum protein 29 (ERp29), which could regulate a group of genes related with cell survival and apoptosis including caspase and Erk and improve locomotor function in spinal cord transection rats.^48^ Furthermore, minocycline, antibody blockade of the CD95 (FAS) ligand and the blockade of glycosphingolipid‐induced iNOS have already proved to suppress the apoptosis of neurons and glia cells, and it is accompanied by improvement in neurological outcomes, and increase the efficacy of cell transplantation strategies.^49^ In another recent study, CARD6, Caspase recruitment domain family member 6, was shown to be a key molecular which could inhibit apoptosis by decreasing Cyto‐c release to cytosol from mitochondria and inhibition of Caspase‐3 signalling pathway.^50^ Zhang et al firstly performed a rat model of SCI, and upregulated p38 was related to inflammation and apoptosis after SCI. Zhang et al also suggested that p38 inhibitor SB203580 treatment could alleviate secondary SCI by suppressing inflammation and apoptosis.^51^ The deficiency of progranulin, a 593 amino acid secreted glycoprotein, is not conducive to SCI recovery via promotion of cellular apoptosis and neuroinflammation.^52^ In a recent study, metformin was found to increase the expressions of β‐catenin and brain derived neurotrophic factor (BDNF), inhibit neuron apoptosis and inflammatory response, and promote motor functional recovery in rats after SCI.^53^

In the above research results, it has been shown that apoptosis contributed to the tissue damage following SCI. Finding the way to inhibit apoptosis after SCI may have important clinical implications for the further treatment. Although more and more studies focus on the intrinsic or extrinsic signals which are associated with apoptosis following SCI, there are more problems still need to be studied, including the detailed mechanisms causing apoptotic death of neurons, astrocytes, oligodendroglia and microglia after SCI.

## 3 THE ROLE OF NECROPTOSIS IN SPINAL CORD INJURY

Apoptosis was known as the only form of PCD traditionally, however, more and more studies showed that necrosis can be induced in a similar manner to apoptosis, which is termed ‘programmed necrosis’ or ‘necroptosis’.^54^ Necroptosis can be regulated by the caspase‐independent pathway and shows the morphological characteristics of necrosis (early loss of plasma membrane integrity, gain in cell volume and swelling organelles).^55^, ^56^ In recent years, necroptosis has been found to contribute to many diseases including neurodegenerative diseases, ischaemic brain injury and viral myocardial infarction.^56^, ^57^, ^58^, ^59^

The most widely studied pathway causing necroptosis results from binding of Tumour necrosis factor‐α (TNF‐α) to TNF‐R1.^60^ Various studies have shown that necroptosis are involved in intracellular signalling cascade transduced by receptor‐interacting protein 1/3 (RIP1/3) and mixed lineage kinase domain‐like protein (MLKL).^61^ RIP1 kinase activity has been verified to be essential for the activation of necroptosis.^62^ In recent research, the author found that overexpress and knockdown PSMB4 could regulate the MLKL and RIP3 pathway in TNF‐α induced necroptosis cell model.^63^ Furthermore, Wang et al found that necrostatin‐1 (Nec‐1), inhibitor of necroptosis, could improve and protect the physiological function by inhibiting necroptosis via inhibition of RIP1/3–MLKL recruitment.^64^ In another research, the results also revealed that necroptosis could contribute to neural cell death, and Nec‐1 could reduce histopathological and functional deficits after SCI in mice.^65^ Therefore, Nec‐1 could be a potential target for treating SCI.

Moreover, accumulating evidences revealed that necroptosis is bound up with inflammation following SCI.^66^, ^67^ Fan et al found that endoplasmic reticulum of necroptotic microglia/macrophages could be manipulated for regulating inflammation following SCI.^68^ Then their subsequent study showed that reactive astrocytes could undergo M1 microglia/macrophages‐induced necroptosis through TLR/MyD88 signalling pathway.^69^ Shao et al found that Smurf1 could facilitate necroptosis of neurons after LPS‐induced neuroinflammation, and Smurf1 may be a potential target for treatment.^70^

Necroptosis was already considered as a critical and emerging mode of PCD following SCI. It has been previously demonstrated that activation of necroptosis leads to neuronal and glial cell death after SCI. Activation of necroptosis has been found to lead to cell loss and tissue damage in central nervous system injury, including SCI. Although some work has been done to explore the molecular mechanism of necroptosis after SCI, the understanding of necroptosis is still inadequate. Therefore, identification of the role of necroptosis in the pathological processes of SCI is necessary to find the therapeutic target for secondary injury.

## 4 THE ROLE OF AUTOPHAGY IN SPINAL CORD INJURY

The word ‘autophagy’ originally came from the Greek and means to eat (‘phagy’) oneself (‘auto’).^71^ Autophagy is known as a non‐apoptotic form of PCD that ensures the maintenance of cellular homeostasis via the degradation and recycle of damaged organelles, toxic agents and long‐lived, needless proteins through an autophagosomal–lysosomal pathway.^72^, ^73^, ^74^ During the course of autophagy, autophagosomes, which are double‐membrane vesicles, sequester cytoplasmic components including damaged organelles and toxic protein aggregates, then the autophagosomes can be combined with lysosomes to make lysosomal proteases degrade the cargo.^75^ Previous studies have revealed that autophagy can promote cell death by inducing caspases‐dependent apoptosis in various cells.^18^, ^76^, ^77^, ^78^ The PI3K/Akt/mTOR is considered as the central signalling pathway involved in autophagy.^79^, ^80^ Elevated autophagy has been observed after SCI and accumulating evidences has revealed that autophagy can act as a pro‐survival mechanism by modulating the death of neural cells for neuroprotection after SCI.^75^, ^81^ Autophagosomes also have been found in cultured neurons or in vivo.^82^, ^83^, ^84^ However, the role of autophagy in the subsequent neurodegeneration following SCI still remained controversial up to now, and both beneficial and detrimental outcomes are reported in previous studies about SCI.

Some researchers believe that autophagy plays an active role in confronting with stress after SCI, and it was considered as a potential therapeutic target of SCI. He et al demonstrated that, in cultured CNS neurons, boosting autophagy could stabilize the microtubules by degrading a microtubule destabilizing protein, SCG10 (superior cervical ganglia protein 10), and this was beneficial to promote axon growth. Moreover, Tat‐beclin1, a specific autophagy‐inducing peptide, could attenuate axon retraction, promote axon regeneration and improve functional recovery in mice with SCI.^85^ Previous study demonstrated that the metformin have a protective effect on SCI which was through autophagy flux stimulation.^86^ Furthermore, autophagy is thought to protect the neurons against endoplasmic reticulum (ER) stress, and its disruption after SCI could lead to ER‐stress‐induced neuronal apoptosis.^87^ Recent studies showed that induction of astrocyte autophagy flux could enhance the viability of neurons, decrease neuronal apoptosis, and improve neurological outcomes.^88^ The relation between autophagy and apoptosis was also discussed in some studies. The results of a previous research showed that induction of autophagy can produce neuroprotective effects in acute SCI in rats via inhibition of apoptosis.^89^ Wang et al proved that autophagy may play a protective role against apoptosis in spinal cord neurons with mechanically‐injury.^90^ Therefore, autophagy may be beneficial for the survival of neurons after SCI.

The other researchers, however, hold a different view. Bisicchia et al found that the inhibition of autophagosomes biogenesis could significantly attenuate remote degeneration and promote spontaneous functional recovery of SCI.^91^ Furthermore, light chain 3 (LC3) is known as an important marker of autophagy, and a previous study proved that the number of the LC3‐positive cells increased significantly at the lesion site after hemisection of the spinal cord, and this result demonstrated that autophagic cell death could be commonly found in the damaged neural tissue following SCI.^81^ In a recent study, ABT888, an inhibitor of Poly(ADP‐Ribose) Polymerase, was found to play a protective role after SCI through a reduction in the autophagy machinery.^92^

Therefore, the effects of autophagy on the different period of SCI are still uncertain, and both protective and pathological functions of autophagy are possible. Therefore, in the future study, we should try to understand the mechanisms in more detail, and be fully aware of the role of autophagy in SCI may carry us a potential pleiotropic treatment that could target multiple cell types and pathways during the pathological process of SCI.

## 5 THE ROLE OF FERROPTOSIS IN SPINAL CORD INJURY

Ferroptosis is known as a newly discovered form of PCD and results from the accumulation of iron‐dependent lipid peroxide, and the term ‘ferroptosis’ was first used by Stockwell et al in 2012.^9^ Ferroptosis is genetically, morphologically and biochemically differ from apoptosis.^93^ Ferroptosis has the characteristic of iron‐dependent increase in reactive oxygen species (ROS), and ROS plays a critical role in ferroptosis.^93^, ^94^ Ferroptosis is considered to be involved in the pathological cell death associated with Alzheimer's diseases, Huntington's diseases, Parkinson's diseases, stroke, traumatic brain injury, ischaemia‐reperfusion injury and so on.^95^ In recent years, more and more studies focus on the role of ferroptosis in SCI.

Ferroptosis is closely related to excitotoxicity‐induced neuron death.^93^ Furthermore, previous study also showed that the neurons in forebrain were more likely affected by ferroptosis, and ferroptosis may be a critical neurodegenerative mechanism in some diseases including Alzheimer's disease.^96^ Glutathione peroxidase 4 (GPX4) is a disincentive for ferroptosis, and ferroptosis inhibition by GPX4 is necessary for the health and survival of motor neuron in vivo.^97^ Because Iron deposition is a key pathological event in ferroptosis, so deferoxamine (DFO), a drug used for treating iron overload, has been used as ferroptosis inhibitors in many disease models.^98^ Previous study found that deferoxamine, a drug used for treating iron overload, could decrease total iron ion, tumour necrosis factor‐α, interleukin‐1β and caspase‐3 expression levels after SCI and inhibit apoptosis and formation of glial scar to improve function recovery.^99^ Furthermore, a recent study focus on the effects of proanthocyanidins on SCI repair, and the results showed that intraperitoneal injections with proanthocyanidins could promote functional recovery of SCI via inhibiting ferroptosis.^100^

These above studies showed that ferroptosis may play an important role in the serious consequences of secondary injury following SCI, and regulating this process contributed to function recovery after SCI. However, adequate studies on the role of ferroptosis in SCI are still lacking in this field. Therefore, research on ferroptosis is urgently needed to unravel the contribution of ferroptosis to neuronal demise and glial scar formation, and this may provide new therapeutic opportunities for SCI.

## 6 THE ROLE OF PARAPTOSIS AND PYROPTOSIS IN SPINAL CORD INJURY

Paraptosis and pyroptosis are two novel types of PCD, and they attract more and more researchers’ interest. Paraptosis has recently been implicated as a type of PCD which has the characteristic of dilation of mitochondria and/or ER swelling resulting from cytoplasmic vacuolation, and it does not depend on caspases and lacks apoptotic morphologies.^101^ Paraptosis lacks the features of apoptosis and it is usually related to defective proteins in the ER, and previous study found a paraptotic response after what appears to involve nuclear targeting.^102^, ^103^ Some studies showed that mitogen‐activated protein kinases (MAPKs) might have key effects on paraptosis, and paraptosis can be supressed by AIP‐1/Alix and cycloheximide.^104^, ^105^ Furthermore, bortezomib/nutlin‐3 could perturb proteostasis, trigger ER/mitochondria stress and irrecoverable impairments in their structure and function, and ultimately lead to paraptotic cell death.^106^ However, as a novel type of PCD, the determinants and consequences of paraptosis in SCI are not clear.

In CNS, it has been found that upregulation of p44 can activate IGF‐1R and neuronal death via paraptosis and autophagy.^107^ In Alzheimer's disease, a previous study found that paraptosis was demonstrated in the early pathological stages of Alzheimer's disease, which might do harm to the mitochondria and lead to mitochondrial pathway‐mediated apoptosis, subsequently.^108^ Furthermore, activated microglia can cause neuronal cell death with characteristic vacuolation following blocking the caspase cascade.^109^ Microglia are known as a key component of the protective scar that forms after SCI, so in‐depth study on the role of activated microglia in paraptosis should be an interesting new direction. In summary, although some progresses have been made in the study of paraptosis, there are still many problems to be solved. Furthermore, the studies of the effects of paraptosis on SCI are insufficient, so there is an urgent need to identify the role of paraptosis in SCI.

Pyroptosis is known as a pro‐inflammatory form regulating cell death which results from caspase‐1 activation within the inflammasome complex and caspase‐11 (caspase‐4/5 in humans) activation following intracellular LPS recognition.^110^, ^111^ Pyroptosis mainly occurs in professional phagocytes of the myeloid lineage including macrophages, dendritic cells and neutrophils, although it has also been observed in keratinocytes, epithelial cells and neurons.^112^ Pyroptosis has been found to make crucial contributions to inflammatory and anti‐microbial responses in infections.^112^ Previous studies showed that the activation of NLRP1 could generate a functional caspase‐1‐containing inflammasome in vivo to regulate the pyroptosis, which has an important effect on the pathogenesis of neurological disorders,^113^ and the rats with NLRP1 or caspase‐1 silencing could result in significantly reduced neuronal pyroptosis in the amygdala kindling‐induced rat model.^114^ In a recent study, the results showed that microglial voltage‐gated proton channel deficiency could reduce NLRP3‐induced neuronal pyroptosis.^115^ Furthermore, the neuroprotection of erythropoetin (EPO) against sevoflurane induced‐neuronal pyroptosis is related to Erk1/2‐Nrf2/Bach1 signal pathway.^116^ A previous study demonstrated that *S pneumoniae* could induce pyroptosis in murine microglia and that NLRP3 inflammasome is critical for caspase‐1 activation during pyroptosis.^117^

The involvement of pyroptosis in the pathological process of SCI has not yet been fully investigated. According to the previous study, pyroptosis has been shown to be closely associated with inflammation, so the relationship between pyroptosis and inflammatory response after SCI deserve our further in‐depth exploration.

## 7 CONCLUSION AND PERSPECTIVE

**FIGURE 1:**
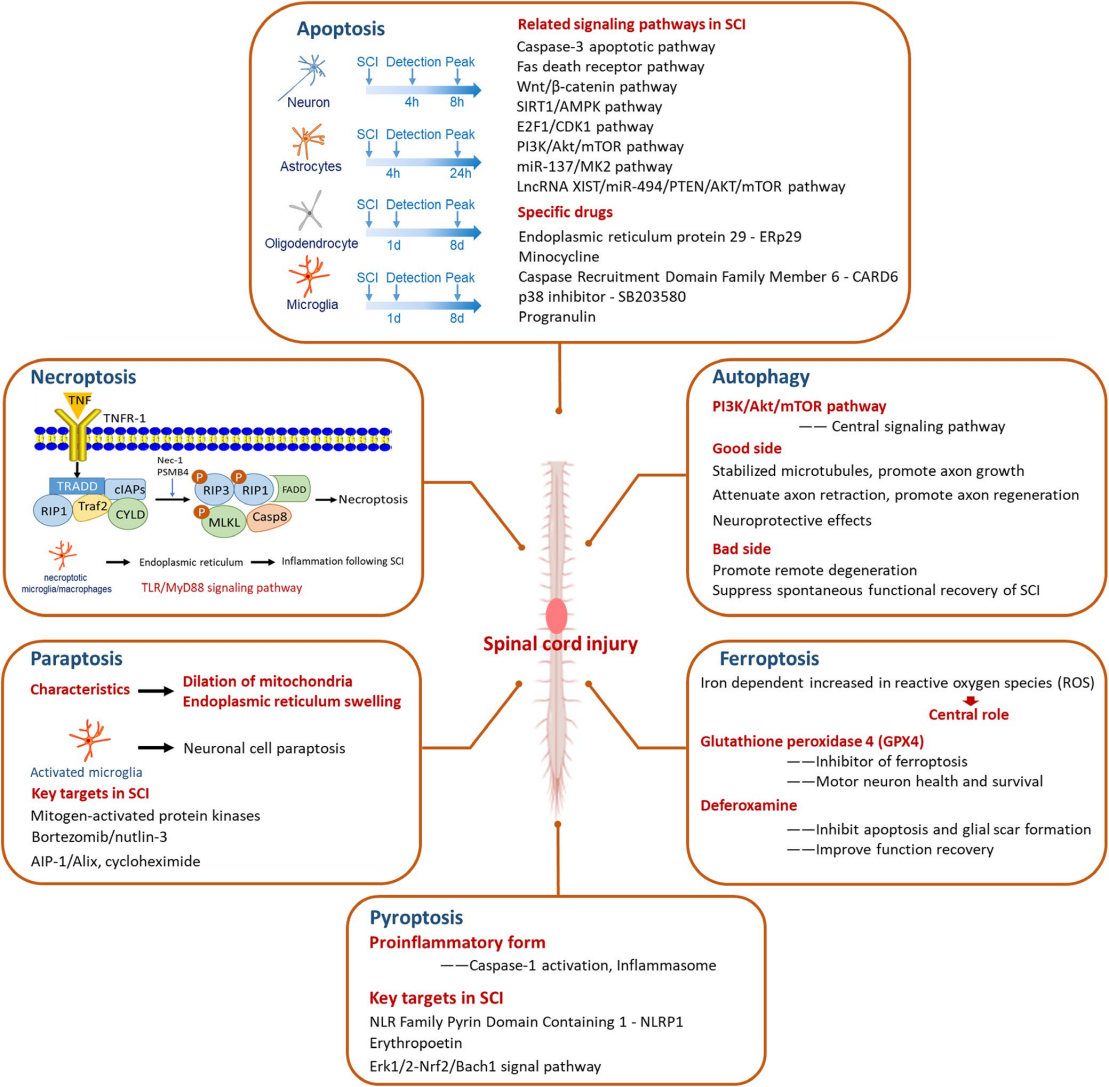
Different types of programmed cell death play different roles in spinal cord injury

SCI and its devastating consequences brings more challenges to clinicians. Therefore, understanding the molecular basis of SCI may be beneficial for improved neuronal, glial survival and neurological deficits. PCD following primary SCI may has critical effect on the secondary injury mechanisms that produce the ultimate neurological deficit. In this review, we briefly summarized the relevant studies that discussed the involvement of PCD in SCI (Figure 1). We conclude that: (a) PCD, including apoptosis, necroptosis, autophagy, ferroptosis, pyroptosis and paraptosis, is generally involved in SCI, but the molecular mechanisms need further investigation; (b) insufficient experiments have confirmed the protective or pathological correlation between PCD and SCI, and this should be further explored; (c) determining the level of PCD in the patients with SCI might be potential tools for its diagnosis, treatment and prognosis.

Based on these conclusions, we make some suggestions concerning the future studies: (a) the correlation among apoptosis, necroptosis, autophagy, ferroptosis, pyroptosis and paraptosis in SCI should be investigated, comprehensively, and it is essential to illuminate which type of PCD play the dominant role in SCI; (b) the relationship between different types of PCD and other molecular mechanisms underlying SCI should be explored; (c) the regulation ways of PCD in SCI should be explored, especially the ways to regulate the expression levels of PCD‐associated genes. (d) the identification of new agents targeting specific PCD pathways in different phases of SCI is needed in the future work.

Although it has been shown that PCD play important roles in different models of SCI, we stress that the complexity of central nervous system injury cannot be reduced to a single physiopathological mechanism or to inhibition or activation of a single cell death type. Therefore, specific combination therapies targeting several PCD modalities could be a more promising strategy for the treatment of SCI. Furthermore, more effort on translating the current findings to post‐injury administration of drugs to block or activate cell death modalities is necessary.

In conclusion, this review can help us to understand the various functions of PCD in the pathological process of SCI. Thoughtful consideration and more detailed findings about the roles of PCD will contribute to our novel understanding of SCI of unknown aetiology in the near future.

## CONFLICT OF INTEREST

The authors declare that they have no competing interests.

## AUTHOR CONTRIBUTIONS

ZS and SF designed the review, ZS, SY, JL and LS collected the articles, ZS wrote the paper, ZS drafted the figures, ZS, GN, XK and SF revised the manuscript. All authors reviewed the final version of manuscript.

## Data Availability

The data of this study are available from the corresponding author upon reasonable request.
